# The primary application of indocyanine green fluorescence imaging in surgical oncology

**DOI:** 10.3389/fsurg.2023.1077492

**Published:** 2023-02-17

**Authors:** Zhang-Yi Dai, Cheng Shen, Xing-Qi Mi, Qiang Pu

**Affiliations:** ^1^ Department of Thoracic Surgery, Sichuan University West China Medical Center, Chengdu, China; ^2^ Western China Collaborative Innovation Center for Early Diagnosis and Multidisciplinary Therapy of Lung Cancer, Sichuan University, Chengdu, China

**Keywords:** indocyanine green, fluorescence imaging technology, near-infrared fluorescence imaging, image-guided surgery, oncology surgery

## Abstract

**Background:**

Indocyanine green (ICG) is a nontoxic, albumin-bound, liver-metabolized fluorescent iodide dye that has been widely utilized in clinical applications since the mid-1950s. However, after the 1970s, in-depth research on the fluorescence properties of ICG greatly expanded its application in the medical field.

**Methods:**

In our mini-review, we searched the relevant literature on common oncology surgeries from PubMed, including lung cancer, breast cancer, gastric cancer, colorectal cancer, liver cancer, and pituitary tumors, using keywords such as indocyanine green, fluorescence imaging technology, and near-infrared fluorescence imaging. In addition, the application of targeted ICG photothermal technology in tumor therapy is briefly mentioned.

**Results:**

In this mini-review, we analyzed studies on ICG fluorescence imaging in common surgical oncology and offered a thorough analysis of each form of cancer or tumor.

**Conclusion:**

ICG has demonstrated significant potential in the detection and treatment of tumors in current clinical practice, although many applications are still in the preliminary stages, and multicenter studies are still required to more precisely define its indications, effectiveness, and safety.

## Introduction

Since the mid-1950s, indocyanine green (ICG) has been utilized in clinical practice. Initially, ICG was used to quantitatively measure hepatic function and cardiac output (1). After the 1970s, studies focused on its fluorescent properties expanded the use of ICG to many other surgical fields, including retina and choroid imaging in ophthalmology (1), cerebral angiography in neurosurgery and cardiac vessel angiography in coronary surgery (2). From the mid-1990s to the early 2000s, the improvement of digital imaging resolution broke through technical barriers and further promoted the widespread development of ICG fluorescent angiography and lymphography (1, 2). As a result, ICG has been developed for many off-label applications in abdominal surgery, thoracic surgery, plastic surgery, lymphatic surgery, pediatric surgery and the detection and treatment of tumors (2–4). Recently, ICG fluorescence imaging has been extensively utilized in cancer-related research, such as sentinel lymph node (the first station of lymph node for malignant tumor drainage) biopsy, tumor locating, imaging-guided surgery procedures, determination of resection margins, lymph node fluorescence harvesting, and anastomotic perfusion assessment. Further clinical studies to explore other emerging uses of the dye are currently ongoing (5).

In this article, we discuss the primary application of ICG fluorescence imaging in common surgical oncology, mainly focusing on sentinel lymph node biopsy (SLNB), tumor locating, imaging-guided surgical procedures, and targeted phototherapy. Finally, in the discussion, we conclude with a brief overview of the future prospects of ICG-guided surgical oncology.

## ICG biochemical and fluorescent profile

ICG is an amphiphilic, water soluble, tricarbocyanine dye (mass = 751.4 Da) that can be applied for intravenous injection (6). In blood, 98% of ICG is bound to plasma proteins, chiefly plasma albumin. The lipophilic component of ICG specifically interacts with plasma proteins by binding to their hydrophobic regions. This structure can produce a nontoxic interface and decrease the eruption of the dye (7). Therefore, ICG is almost nontoxic unless the patient is allergic to iodide (5). The hepatocyte takes up the dye *via* carriers and clears up more than 80% of the available ICG into the biles within 18 h of administration (2, 6), a process that follows first-order metabolism with matching uptake and clearance rates. In the first 10–20 min after injection, ICG is cleared out of the blood into the bile with a half-life of 3–4 min (7). However, after the initial period, the rate of clearance decreases gradually, and residual ICG can remain in the blood for more than 1 h (2, 7).

ICG is excited by a laser with a wavelength between 750 and 800 nm, and its fluorescence peaks at approximately 830 nm, which allows up to 10–15 mm of penetration into tissues and avoids autofluorescence from endogenous tissues (2). Therefore, ICG fluorescence (ICG-F) has high contrast and sensitivity (5). Moreover, ICG is inexpensive and readily available, promoting its application in the medical field, especially in surgery.

## ICG in used sentinel lymph nodes (SLNs) biopsy of common surgical oncology

The SLN receives the first lymphatic flow from malignant tumors, and it is universally the first site of detection for potential malignancy dissemination (8). In surgery, SLNs are often difficult to detect or dissect completely during surgery.

ICG near-infrared (NIR) imaging technology to detect SLNs and visualize lymphatic vessels was introduced by Lim and Soter (9) in 1993. ICG offers unparalleled advantages in lymphatic drainage, especially the detection of deeper lymphatic structures, which is more accurate than technetium-99 and methylene blue (MB). This is especially important for obese patients (10). Moreover, ICG also avoids the risk of radiation exposure (11, 12). Table 1 summarizes the ICG dosage and administration in the studies mentioned in this subsection.

**Table 1:** Characteristics of studies evaluating the ICG technique of SLNB in different cancers.

SLN biopsy	Study	Year	Dose	Route	Number of cases	Success (%)	False-negative
Lung cancer	Ito	2004	25 mg	Peritumoral	38	87.5	1/38
	Yamashita	2011	10 mg	Peritumoral	61	80.3	1/49
	Yamashita	2012	10 mg	Peritumoral	31	80.7	0/24
	Nomori	2016	10 mg	Peritumoral	135	84.0	1/3
	Kawamkami	2020	2.5–10 mg	Peritumoral	22	72.7	1/13
Breast cancer	Kitai	2005	25 mg/5 ml	Subareolar	18	94	--
	Guo	2017	1.25 mg	Subareolar	200	97	--
	Jung	2019	0.6 mg/1 ml	Subareolar	58	94.7	--
	Zhang	2021	1.25 mg	Subareolar	197	96.9	--
SLN biopsy	Study	Year	Dose	ICG administrati on	Enrolled studies	Detection rate (%)	Sensitivity (%) (metastasis in SLN/total lymph node metastasis)
Colorectal cancer	Catalin Alius	2020	0.5–5.0 mg/ml (0.5–5.0 ml)	Subserosal/submucosal	12	90.0–95.0	0.0–85.6
Gastric cancer	Huang	2021	No reporting	Subserosal/submucosal	54	99 (95% CI: 97%–99%)	90 (95% CI: 82%–95%)

ICG, indocyanine green, SLN, sentinel lymph node. Success was defined as the ability to detect sentinel lymph nodes. False-negative: *n* of true positive nodes/*n* of total negative nodes.

The use of nonfluorescent tracers in sentinel lymph node biopsy (SLNB) of lung cancer has had limited success in the past (13). In recent years, ICG has been injected into the peritumoral area for SLNBs in lung cancer, reporting a varied detection rate. Ito et al. (14) were the first to report that ICG was used for SLNB in lung cancer. A suspension of 5 ml (5 mg/ml) ICG and 400 U hyaluronidase was injected into the peritumoral area to identify SLNs, and the results showed a positive predictive value of 97% and a sensitivity of 87.5%. In this article, SLNs were defined as ICG concentrations greater than 1.25 times the average of all lymph nodes (LNs). However, this is arbitrary and questionable and lacks supporting data. In the studies of Yamashita et al. (15, 16), 10 mg ICG was similarly injected around the tumor in 61 patients. Subsequently, the anesthesiologist inflated and deflated the lung several times, and after 10 min, LNs were inspected under fluorescence imaging technology (FIT). This method achieved a lower SLN detection rate (80.3%) and false-negative rate (2.1%). Nomori (17) and Kawakami et al. (18) also used a peritumoral injection of ICG (2.5–10 mg) for SLNB, 84% and 72.7% of which found SLNs, respectively. However, in the study of Nomori et al. (17), the false-negative rate was over 20%, which may be associated with the rapid hepatic clearance of ICG and mechanical reasons such as pleural adhesions and fissure hypoplasia. If a fluorescent signal is detected in the N2 (mediastinal) LN stations but N1 (hilar and more distal) LN stations are not, this phenomenon in lung cancer is called “skip” metastasis. These studies demonstrated the feasibility of detecting SLNs after peritumoral injections of ICG, but no studies have been able to accurately identify “skip” metastatic LNs.

Different ICG concentrations, injection timings, solidity ratios of nodules, and mechanical issues may contribute to the detection rate of SLNs. In the study of Phillips et al. (19), ICG was injected into the peritumoral area to perform SLNB under FIT. The SLN(s) detection rate was significantly increased with ICG dose ≥1 mg versus ICG < 1 mg (65.2% vs. 35.0%, *P* = 0.05), albumin dissolvent versus fresh frozen plasma and sterile water (68.1% vs. 28.6% vs. 20%, *P* = 0.01), postinjection lung ventilation (65.2% vs. 35.0%, *P* = 0.05), and solid nodules versus subsolid nodules (77.4% vs. 33.3%, *P* = 0.04).

In breast cancer, surgeons who used ICG-FIT to perform SLNBs reported a 94% (17/18) detection rate (20). According to Jung et al. (21), the SLN detection rate of the dual method (DM) group, which included ICG-F and radioisotope (RI), was 98.3%, as opposed to 93.8% in the RI alone group, although there was no significant difference between the two groups (*P* = 0.14). Zhang (22) and Guo et al. (11) also demonstrated that the DM of ICG and methylene blue (MB) was significantly higher than that of MB alone (96.9% > 89.7%, *P* < 0.05; 99.5% > 89%, *P* < 0.001). ICG-FIT has the advantages of shorter imaging intervals between injection and visualization of lymphatic vessels and does not require a gamma probe compared to traditional tracers of technetium-99 or MB (23). Combining ICG and other traditional tracers is more effective than RI or MB alone in detecting SLNs. Larger, randomized controlled trials are needed to confirm these conclusions.

ICG NIR imaging technology is also used in gastrointestinal surgery research. Catalin Alius et al. (10) analyzed a total of 337 patients enrolled to explore the efficiency and sensitivity of ICG NIR in SLNBs in colon cancer surgery. The results showed that the detection rate of ICG was up to 90%–95%; thus, they considered it to be useful in lymphography and locating SLNs in colorectal cancer. Huang et al. (24) reported a combined SLN detection rate, accuracy, and sensitivity of 99% (95% CI: 97%–99%), 98% (95% CI: 95%–99%), and 90% (95% CI: 82%–95%), respectively, in cases of gastric cancer (GC). However, clinical studies have also shown that the sensitivity (ranging from 0% to 85.6%) of ICG to identify nodal metastases varies due to the variable methods used and the heterogeneity of the study groups (10). In addition, some studies have shown that ICG might elevate the efficiency of regional LN dissection (25–27) and increase the total number of LNs retrieved for patients with GC in three aspects (28). Meanwhile, the perioperative risk for patients did not increase. Zhong et al. (29) analyzed the figures of two randomized controlled trials and showed that the average number of LNs retrieved in the ICG group was higher than that in the non-ICG group (49.9 vs. 42.0, *P* < 0.001). However, Park et al. (30) found that ICG imaging was not recommended for selective LN dissection in advanced GC because of the limited staining of perigastric LNs. To assess whether ICG-F imaging has a survival benefit in patients with different stages of GC, additional high-quality randomized controlled trials with larger sample sizes are needed (31, 32).

## ICG in lung cancer

With the popularity of higher-resolution spiral computed tomography (CT), the detection of ground-glass opacities (GGOs), which are often pathologically proven to be early-stage lung cancer, is becoming increasingly common. Thus, surgeons are more likely to be exposed to this clinical condition than ever before. Identifying and locating the anatomical locations of small nodules and GGOs intraoperatively is not simple, especially in minimally invasive surgery. Identifying and locating GGOs intraoperatively is a major application of ICG NIR imaging in thoracic surgery. ICG is usually injected into the peritumoral area under CT or electromagnetic navigation bronchoscopy (ENB) guidance and then visualized by an NIR fluorescence imaging system. An average of 15 mg ICG was injected into peripheral lung nodules by Ujiie et al. (33) under the guidance of CT. Eighteen of 20 (90%) nodules were localized exclusively under NIR fluorescence thoracoscopy. One non-locatable nodule was 4.8 cm from the parietal pleura, and the other was undetected due to a technical problem of ICG delivery. In the study of Abbas et al. (34), 5–10 mg ICG was injected into the peritumoral area under ENB in 30 patients for tumor detection, followed by surgical resection. They reported an overall success rate of 98.1% (34). In a retrospective study conducted by Zhang et al. (35), patients who had small pulmonary nodules underwent lung resection after localization with the guidance of ENB. The success rate of nodule localization was 98.3% (178/181), while the accuracy was 89% (161/181) without any complications. To assess the feasibility and safety of intraoperative NIR fluorescence imaging for the detection of GGOs, clinical trials were conducted by Okusanya (36) and Kim et al. (37). They concluded that ICG-FIT could safely find hidden pulmonary nodules, which are difficult to find in CT or finger palpation. In addition, as little as 1 mg/kg ICG is sufficient for surgeons to identify and locate pulmonary nodules. However, it is worth noting that ICG was administered intravenously in their studies, not directly into the peritumoral area. Moreover, both studies showed that the depth of the GGOs from the pleural surface played a considerable role in the ability of FIT to detect nodules without transecting the parenchyma above the nodule. Although ICG-FIT has a high success rate in identifying pulmonary nodules, it is worth noting that ICG is a sensitive but not specific contrast agent because it is unable to distinguish tumors from inflammation.

## ICG in gastrointestinal cancer

The application of ICG in gastric cancer (GC) resection began more than 20 years ago. Initially, ICG was used for SLNB and anastomotic blood flow perfusion (38). At present, the application of ICG-FIT in laparoscopic radical gastrectomy is still in the initial stage of clinical practice. Controversy remains regarding its safety and efficacy.

Accurate identification of tumor sites and boundaries often relies on the surgeon’s visual experience in the past. During laparoscopic surgery for GC, however, identifying the tumor location purely by visual experience is difficult and inadequate, and commonly used methods, including endoscopic suture or clamp marking, have multiple drawbacks (39). Moreover, tumors located inside the stomach or intestine are challenging to detect due to a lack of tactile sensation. Ushimaru et al. (39) retrospectively analyzed ICG (*n* = 84) and non-ICG (*n* = 174) groups according to whether they received preoperative injection of ICG into the mucosa and found that ICG-FIT was practicable and safe and could be used as a tumor marker to determine the resection margin. Yoon et al. (40) analyzed a total of 93 patients who underwent laparoscopic distal gastrectomy (non-ICG group, *n* = 70) or laparoscopic distal gastrectomy using ICG (ICG group, *n* = 23), and they demonstrated that ICG injection for securing the proximal resection margin in totally laparoscopic distal gastrectomy was a safe and accurate oncology procedure. Lee et al. (41) designed an ICG-loaded alginate hydrogel as a fluorescence surgical marker and performed precise laparoscopic operations in porcine models. The results indicated that even after 3 days, the real-time injection sites of the hydrogel could be accurately detected in the porcine stomach. However, to date, there is no uniform standard for the injection dose and site of ICG in gastric cancer surgery. Different ICG injection methods, as well as advanced stages, may influence fluorescence efficacy (27, 28).

In colorectal cancer surgery, ICG can be used for various functions, such as tumor fluorescence localization, LN fluorescence harvesting, intraoperative angiography and anastomotic leakage (AL).

Laparoscopic localization of early colorectal tumors remains challenging for surgeons because of the lack of tactile sensation (42). In light of ICG fluorescence imaging systems, surgeons can easily localize early-stage colorectal cancer, especially at sites that cannot be easily identified through the serosa surface after endoscopic mucosal resection or submucosal dissection (43). Another application is LN fluorescence imaging, which is described in detail in section 3.3. ICG is injected into the submucosal or subserosal layer, and the lymphatic pathway can be intactly and clearly visualized under the NIR imaging system (44). In addition, intraoperative ICG angiography (ICGA) could be used to determine the optimal cutting plane by angiography before colon transection, preserve the perfusion of the anastomosis to the greatest extent, and prevent the complications of ischemia-related AL (45). However, the efficiency of intraoperative ICGA to prevent ischemia-related AL remains controversial. A meta-analysis by Pang et al. demonstrated that ICGA significantly reduced the AL rate (OR = 0.33; 95% CI: 0.24–0.45; *P* < 0.0001) after rectal cancer resection (46). The AL rate was much lower in the ICG group (3.5%) than in the no-ICG group (10.5%) (*P* = 0.041) in a retrospective study by Ishii et al. (47). However, a multicenter randomized controlled trial by De Nardi et al. (48) demonstrated that there was no statistically significant reduction in the AL rate in the ICG group. The reason may be associated with the different characteristics of the blood supply of the colon and rectum. ICG is used in a variety of ways in colorectal surgery, but the positive clinical results are frequently unsatisfactory, and more high-quality, large-scale randomized controlled trials are needed.

## ICG in liver cancer

Some attempts have been made to detect hepatocellular carcinoma (HCC) *via* ICG by Ishizawa et al. (49). They initially used ICG to measure liver function preoperatively, and then ICG fluoroscopy was used to detect HCC in laparoscopic hepatectomy. Two clinical trials in 2009 showed that ICG image navigation was extremely sensitive in detecting small, difficult-to-identify liver tumors (50, 51). All liver tumors, including metastases, were well identified by ICG fluorescence technology, and it is particularly noteworthy that ICG appears to be particularly sensitive to well-differentiated hepatocellular carcinoma (50, 51). Aoki et al. (52) also conducted an experiment to demonstrate that ICG was reliable for identifying hepatic segments and subsegments for anatomical HCC resection by injecting ICG into the portal vein. The demarcation of the liver segment and subsegment was clearly observed in 33 (94.3%) of the 35 patients under the NIR camera system PDE-2 (52). Additionally, the findings of two meta-analyses pointed to an association between ICG-guided laparoscopic hepatectomy and lower complications, shorter hospital stays and surgical durations, and less intraoperative blooding (53, 54). A single-center study from Wang et al. suggested that ICG-guided laparoscopic hepatectomy appears to be associated with better overall survival (55). Kudo et al. (56) revealed that fluorescence imaging could also identify subcapsular liver tumors through the accumulation of ICG after preoperative intravenous injection in cancerous tissues of HCC. Meanwhile, some studies also explored whether recurrent hepatic tumors, including extrahepatic tumors that cannot be identified by routine ultrasonography, could be detected by this novel imaging modality during laparoscopic hepatectomy (57, 58). These studies have demonstrated the potential of ICG NIR imaging to detect extrahepatic metastases from HCC, which exhibit fluorescent signals when illuminated by NIR light, indicating its capability to transport ICG (58).

Residual positive margins after surgery for liver tumors remain a key factor in high recurrence rates. A study by Lieto et al. (59) revealed that ICG could determine the positive residual margins and undiagnosed lesions and improve both the integrity of radical resection and intraoperative staging of primary and secondary liver tumors.

In recent years, ICG-FIT has also been used for detecting hepatoblastomas (HBs) since HBs possess similar features to HCC (60).

## ICG in pituitary tumors

Currently, ICG-guided surgery has been applied to pituitary tumors, and its usefulness has been assessed by many studies. However, their sensitivity and specificity vary across the literature and between functioning and nonfunctioning adenomas (61). The study of Amano et al. (62) demonstrated that the differentiation of fluorescent intensities between both functioning/nonfunctioning lesions and normal glands was clear after intravascular injection of ICG in 15 patients. Other studies employing ICG reported similar findings (63–66). In contrast, in the study of Hide et al. (67), there was little difference between pituitary gland and tumor intensity in 26 patients. Table 2 summarizes the differentiation of fluorescent intensities in each study mentioned above. The reason for the discrepancy in results within the studies may be related to the small sample size. However, there is still a long way to go to assess the effectiveness of ICG-guided surgery in different tumor subtypes and to explore the relationship between its clinical application and possible clinical outcomes.

**Table 2:** Characteristics of studies reporting on the use of ICG in pituitary adenomas.

Author	Year	Samples	Success (%)	Results	Note
Jeon et al. (42)	2019	8	100	Signal with an average SBR of 4.1 ± 0.69.	100% sensitivity but only 29% specificity for both functioning and nonfunctioning adenomas.
Amano et al. (41)	2019	15	100	The intensity of fluorescence of tumor and normal tissues showed clear differences.	These patients had complete removal of their tumor. Preservation of the gland, and restoration of normal endocrine function.
Verstegen et al. (45)	2016	10	50	In 9/10 cases, normal gland exhibited a stronger fluorescent signal than the adenoma, 5/10 adenomas had no fluorescent signal, 3/10 had only weak fluorescence, and 1 had a strong signal.	In 1 patient, no intensity of fluorescence distinction between the normal pituitary gland and adenoma.
Hide et al.	2015	26	Not applicable	The median of the peak color value was 123.0 for the normal gland, and 136.5 for pituitary adenomas.	No significant difference in the time at peak intensity between adenoma and normal gland.
Sandow et al. (43)	2015	22	100	11 tumors were visualized directly; 11 were seen indirectly due to lower ICG fluorescence intensity compared to surrounding tissue.	The fluorescence distinction between the normal pituitary gland and adenoma is clear.
Litvack et al. (44)	2012	12	92 (11/12)	In every case of nonfunctioning macroadenoma, a difference in fluorescence from the normal pituitary gland was noted.	Adenoma was less fluorescent than normal pituitary gland. 1 patient is excluded due to a dye cross-allergy.

SBR, signal-to-background ratio; Success was defined as the ability to detect NIR signal.

## Targeted ICG

Currently, a hot-point of the ICG treatment field is ICG-targeted phototherapy. Photothermal therapy *via* the use of laser light causes thermal injury and apoptosis of targeted neoplastic tissue and is an investigational treatment method. When ICG is reconstituted with antibodies against the epidermal growth factor receptor, it can specifically bind to this receptor on the surface of tumor cells (68). Then, ICG-targeted phototherapy mediates tumor cell apoptosis through laser light of specific wavelengths, and the efficiency is as high as 90%–100% (69). These experiments showed massive potential for novel diagnostic and therapeutic interventions for ICG.

## Discussion

Due to the excellent physical and chemical properties of ICG, it has been widely used in clinical practice. In this work, we reviewed many studies describing the development and use of ICG in cancers. However, many meaningful and interesting works, such as vascular surgery, biliary surgery, and ophthalmology, have been omitted simply because there are many previous overviews that have been detailed (10) and unrelated to our topic of interest. With the development of a myriad of ICG-related equipment and technology, surgeons may benefit from the increasing use of ICGA and ICG lymphography. In addition, ICG can also be used in the intraoperative staging and potential treatment of cancers. However, it is obvious that more studies are still needed to better define its indications, applications, and toxicity.

## Author contributions

Z-YD contributed to the writing of the manuscript, data analysis, and tabulation. X-MQ made revisions to the language and important intellectual content. CS contributed to data interpretation and analysis. QP proposed the concept and design of the study. All authors contributed to the article and approved the submitted version.
